# CD40, a Novel Inducer of Purinergic Signaling: Implications to the Pathogenesis of Experimental Diabetic Retinopathy

**DOI:** 10.3390/vision1030020

**Published:** 2017-08-12

**Authors:** Carlos S. Subauste

**Affiliations:** 1 Division of Infectious Diseases and HIV Medicine, Department of Medicine, Case Western Reserve University, Cleveland, OH 44106, USA; carlos.subauste@case.edu; Tel.: +1-216-368-2785; 2 Department of Pathology, Case Western Reserve University, Cleveland, OH 44106, USA

**Keywords:** diabetes, retina, Müller cells, endothelial cells, inflammation, cell death

## Abstract

Diabetic retinopathy is a leading complication of diabetes. Death of capillary cells with resulting capillary degeneration is a central feature of this disease. Chronic low-grade inflammation has been linked to the development of retinal capillary degeneration in diabetes. CD40 is an upstream inducer of a broad range of inflammatory responses in the diabetic retina and is required for death of retinal capillary cells. Recent studies uncovered CD40 as a novel inducer of purinergic signaling and identified the CD40-ATP-P2X_7_ pathway as having a key role in the induction of inflammation in the diabetic retina and programmed cell death of retinal endothelial cells.

## 1. Introduction

Diabetes is one of the most common chronic diseases in the world. The prevalence of diabetes worldwide in 2014 increased to 8.5% in adults over 18 years of age leading to the estimation that there are 422 million patients with diabetes in the world [1]. Microangiopathy is a major cause of morbidity and mortality in diabetics and the retina is one of the main tissues affected by diabetic microvascular disease. The overall prevalence of diabetic retinopathy (DR) is 35% among individuals with diabetes [2]. Moreover, DR is the leading cause of vision loss among individuals from 20 to 74 years of age in the United States [3].

Retinal vascular pathology is a key cause of clinically significant loss of vision in diabetics. Early vascular lesions of DR include the death of retinal endothelial cells and pericytes [4]. This leads to the transformation of capillaries into tubes of basement membrane devoid of cells (capillary degeneration). Degenerate retinal capillaries lack blood flow [5] and thus contribute to the development of retinal ischemia and subsequent neovascularization in advanced diabetic retinopathy. Neovascularization and macular edema are leading causes of vision loss in DR.

Various mechanisms appear to link chronic hyperglycemia to the development of microangiopathy. These include oxidative stress, increased polyol pathway flux, increased hexosamine pathway flux, activation of protein kinase C and increased formation of advanced glycation-end products [6]. Chronic low grade inflammation also plays an important role in the pathogenesis of DR [7]. ICAM-1 is upregulated in retinal endothelial cells in diabetic retinas from humans and rodents, and ICAM-1 promotes an increase in the number of leukocytes adherent to vessel walls (leukostasis) in diabetic rats [8,9]. Blockade of ICAM-1 diminishes capillary degeneration in diabetic mice [10]. Retinal levels of inducible nitric oxide synthase (NOS2) are increased of patients with DR and in diabetic rodents [11,12]. Moreover, diabetic NOS2^−/−^ mice are protected from retinal leukostasis and capillary degeneration [13,14]. The vitreous fluid in patients with proliferative DR [15] and retinas of diabetic rodents [16] exhibit increased expression of the chemokine (C-C motif) ligand 2 (CCL2, also known as monocyte chemoattractant protein 1 or MCP-1). CCL2 recruits leukocytes including monocytes and dendritic cells to sites of inflammation. The correlation between CCL2 protein levels in the vitreous with the clinical stage of diabetic proliferative retinopathy [17] suggests a pathogenic role for this chemokine.

## 2. CD40 is Required for Development of Experimental DR

Increasing evidence indicates that CD40 is a key upstream regulator of various inflammatory responses in the diabetic retina and a central mediator of the development of experimental DR [18,19,20,21]. CD40 is a TNF receptor superfamily member expressed on various hematopoietic and non-hematopoietic cells [22,23,24]. CD154 (CD40 ligand) is expressed on activated T cells and platelets, and can also be present in plasma as a soluble protein [22,23]. In addition to retinal microglia/macrophages, non-hematopoietic cells including retinal endothelial cells (REC), Müller cells and retinal ganglion neurons have constitutive low-level expression of CD40 [25].

The CD40–CD154 pathway is activated in diabetes: CD40 expression is increased in Müller cells, REC and microglia/macrophages in diabetic mice [20], and blood levels of CD154 are increased in diabetic mice and patients with microangiopathy [21,26,27,28]. Moreover, serum CD154 from diabetics induces pro-inflammatory responses in endothelial cells and monocytes [28]. Studies in diabetic CD40^−/−^ mice revealed that CD40 is central to ICAM-1 upregulation in REC, leukostasis, upregulation of TNF-α, IL-1β and NOS2 retinal mRNA levels, retinal protein nitration and increased CCL2 mRNA in the retina [19,20,21]. In addition, diabetic CD40^−/−^ mice do not develop capillary degeneration indicating that CD40 is critical for early experimental DR [20,21]. In vitro studies also support the importance of CD40 as a key mediator of inflammatory responses in retinal cells. CD40 ligation causes upregulation of ICAM-1, CCL2, NOS2 and PGE_2_ in Müller cells and/or REC [19]. CD40 stimulation in macrophages/microglia increases TNF-α and IL-1β secretion and upregulates NOS2 [29,30,31].

## 3. CD40 is a Novel Inducer of Purinergic Signaling: CD40 Induces ATP Release by Müller Cells Triggering P2X_7_-Driven TNF-α/IL-1β Production by Macrophages/Microglia

Myeloid cells are central mediators of inflammation. However, studies using bone marrow transplants in a mouse model of ischemia/reperfusion-induced retinopathy (a CD40-driven disease) revealed the importance of CD40 expressed on non-hematopoietic cells for the development of ischemic retinopathy. The induction of retinal inflammation with the resulting increase in ganglion neuron loss in this model requires expression of CD40 in non-hematopoietic cells but not in hematopoietic cells [32]. In addition, we recently uncovered a novel mechanism by which CD40 on non-hematopoietic cells (Müller cells) induces by-stander macrophages/microglia to express pro-inflammatory cytokines and promote development of experiment diabetic retinopathy [21,33].

**Figure 1:**
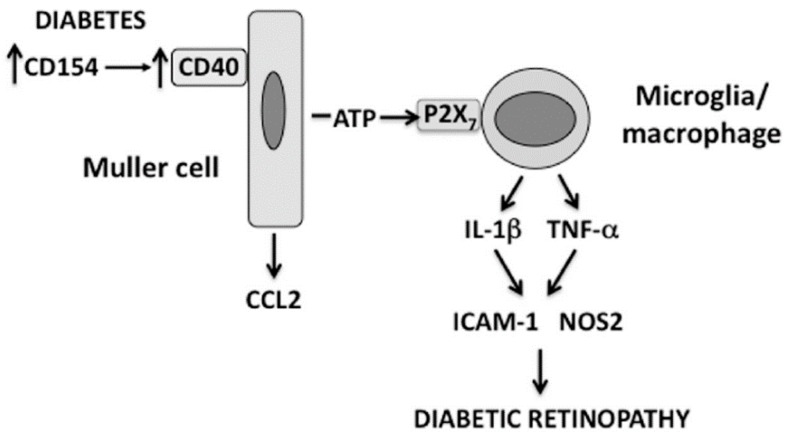
The CD40-ATP-P2X_7_ pathway links cellular responses in Müller cells with the induction of inflammatory responses in bystander microglia/macrophages in DR. Blood levels of CD154 and expression of CD40 on Müller cells are increased in diabetes. CD40 ligation in Müller cells causes CCL2 upregulation and secretion of extracellular ATP. In turn, ATP binds P2X_7_ expressed in microglia/macrophages leading to upregulation of TNF-α and IL-1β as well as ICAM-1 and NOS2, pro-inflammatory molecules that can be induced by these inflammatory cytokines.

Müller cells are the main macroglia in the retina. These cells become dysfunctional in diabetes and contribute to the development of experimental DR [34]. Transgenic mice on a CD40^−/−^ background that exhibit rescue of CD40 restricted to Müller cells (Trg-CD40 mice) were generated to study the role of CD40 expressed in Müller in the pathogenesis of DR [21]. After becoming diabetic, these mice exhibit upregulation of TNF-α, IL-1β, ICAM-1, NOS2 and CCL2 in the retina and develop capillary degeneration (early experimental DR) [21]. Diabetic transgenic mice without rescue of CD40 (Trg-Ctr mice) do not upregulate these pro-inflammatory molecules and do not develop capillary degeneration [21]. Interestingly, in vitro studies revealed that while Müller cells secrete CCL2 in response to CD40 ligation, these cells do not produce TNF-α and IL-1β in response to this stimulation [19,21]. These findings raised the possibility that CD40 stimulation in Müller cells recruits other retinal cells to upregulate TNF-α and IL-1β. Indeed, co-culture experiments of Müller cells and monocyte/macrophages showed that CD40 ligation causes release of extracellular ATP by in Müller cells that in turn triggers TNF-α and IL-1β secretion by monocytes/macrophages that is mediated by the purinergic receptor P2X_7_ expressed on these myeloid cells [21]. Several lines of evidence support the in vivo relevance of the newly discovered CD40-ATP-P2X_7_ pathway in experimental DR. Retinal microglia/macrophages are the cells that upregulate TNF-α protein levels both in diabetic Trg-CD40 mice and in diabetic wild-type (WT) animals [21,35]. Diabetic Trg-CD40 mice treated with the P2X_7_ inhibitor BBG and diabetic P2X_7_^−/−^ mice do not upregulate TNF-α and IL-1β, as well as NOS2 and ICAM-1, molecules induced by TNF-α/IL-1β [21]. Further support of the relevance of CD40-ATP-P2X_7_ pathway in experimental DR comes from the demonstration that both diabetic Trg-CD40 mice and WT mice upregulate P2X_7_ protein levels in retinal microglia/macrophages [21]. This finding is significant because P2X_7_ upregulation is a key feature of P2X_7_-driven diseases and increased P2X_7_ expression in microglia is sufficient to stimulate pro-inflammatory cytokine expression [36]. Taken together, these results suggest that the CD40-ATP-P2X_7_ pathway is a central mediator of upregulation of TNF-α, IL-1β, NOS2 and ICAM-1 in the diabetic retina (Figure 1).

Increase in cytoplasmic Ca^2+^ is a mechanism that can lead to release of extracellular ATP. Recent evidence supports that CD40 stimulation causes release of extracellular ATP through activation of phospholipase Cγ1 (PLCγ1), a molecule that causes Ca^2+^ flux from the endoplasmic reticulum into the cytoplasm increasing cytoplasmic Ca^2+^ concentrations [21]. CD40 ligation in Müller cells causes rapid tyrosine 783 phosphorylation of PLCγ1, a marker of PLCγ1 activation [21]. CD40 stimulation is reported to increase cytoplasmic Ca^2+^ in B cells and smooth muscle cells [37,38]. Moreover, incubation with the calcium chelator BAPTA AM or with the PLC inhibitor U73122 prevents the ability of CD40 ligation to cause release of ATP [21]. These findings support the conclusion that PLCγ1 acts as a molecular link between CD40 and activation of purinergic signaling.

## 4. The CD40-ATP-P2X_7_ Pathway Mediates Programmed Cell Death (*PCD*) of Retinal Endothelial Cells

**Figure 2:**
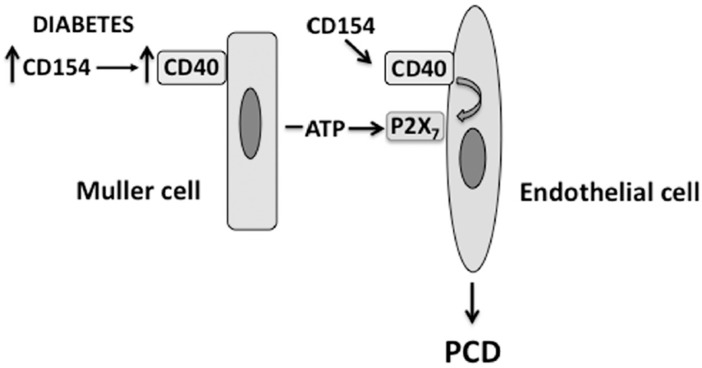
The CD40-ATP-P2X_7_ pathway links cellular responses in Müller cells with programmed cell death (PCD) of bystander REC in DR. Blood levels of CD154 and expression of CD40 on Müller cells are increased in diabetes. CD40 ligation in Müller cells causes secretion of extracellular ATP. At the level of REC, CD40 upregulates P2X_7_ expression, making REC susceptible to P2X_7_-induced programmed cell death.

Death of REC results in the development of degenerate capillaries, an event believed to cause retinal ischemia. Various mechanisms appear to contribute to the development of capillary degeneration. These include increased oxidative and nitrosative stress [12,13,39,40,41] as well as expression of inflammatory molecules (TNF-α, IL-1, ICAM-1) [42,43,44,45]. CD40 is upregulated in REC of diabetic mice and the presence of CD40 is required for development of capillary degeneration. However, CD40 ligation on endothelial cells promotes their survival rather than death. P2X_7_ can induce cell death by apoptosis or necrosis [46]. Indeed, the CD40-ATP-P2X_7_ pathway explains how CD40 can lead to PCD of REC. Müller cells closely associate with REC and, as explained above, secrete ATP in response to CD40 ligation. While REC are relatively resistant to PCD when incubated with extracellular ATP, CD40 ligation in these cells causes P2X_7_ upregulation making them susceptible to ATP-dependent PCD [18]. This mechanism is likely to occur in vivo since P2X_7_ is upregulated in REC from diabetic B6 but not CD40^−/−^ mice, and diabetic CD40^−/−^ and P2X7^−/−^ mice exhibit reduced PCD of REC [18]. Altogether, the CD40-ATP-P2X_7_ pathway appears to be an important driver of PCD of REC in diabetes (Figure 2). Given that primary human pericytes also express CD40 (J-A Portillo and C.S. Subauste, unpublished observations), it is possible that the CD40-ATP-P2X_7_ pathway may also mediate PCD of retinal pericytes in diabetes.

Further support of the importance of P2X_7_ in diabetic microangiopathy comes from the demonstration that induction of diabetes is accompanied by increased susceptibility of retinal microvessels to P2X_7_-mediated death [47,48]. Functional studies suggest that the number of P2X_7_ receptors is not significantly increased in retinal microvessels of diabetic animals [47]. Similarly, fibroblasts exposed to high glucose and fibroblasts isolated from diabetics exhibit increased susceptibility to P2X_7_-mediated cell death through a mechanism that does not require upregulation of P2X_7_ expression [49,50]. Taken together, these studies suggest that CD40-mediated P2X_7_ upregulation and hyperglycemia-induced changes in intrinsic receptor properties may contribute to the increased death of retinal capillary cells in diabetes.

The studies discussed herein uncovered CD40 as a key mediator of DR. It remains to be determined how CD40 is upregulated in diabetes and how CD154 gains access to the retina. The discovery of the CD40-ATP-P2X_7_ pathway provided the first definitive demonstration that an inflammatory disorder can develop when CD40 is expressed exclusively in a non-hematopoietic cell. This finding is likely to have therapeutic implications. CD40 is a recognized target for the treatment of various diseases with an inflammatory component. Signaling pathways downstream of CD40 have different relative roles in the induction of inflammatory responses in hematopoietic vs. non-hematopoietic cells [19,30]. CD40-TNF Receptor Associated Factor 6 (TRAF6) is the signaling pathway by which CD40 induces inflammatory responses in myeloid cells [30,51], but is also central to the activation of various cell-mediated immune responses required for control of intracellular pathogens [30,31,51,52]. Thus, attempts to inhibit CD40 signaling active in myeloid cells have the risk of causing susceptibility to opportunistic infections. While CD40-TRAF6 signaling also promotes inflammatory responses in non-hematopoietic cells, blockade of CD40-TRAF2 signaling is sufficient to markedly impair these responses in non-hematopoietic cells including Müller cells [19,30,53]. Moreover, the CD40-TRAF2 pathway does not play a significant role in the induction cell-mediated immune responses [30,31,51,52]. The demonstration that CD40 signaling in Müller cells activates inflammatory responses not only in Müller cells but also in bystander microglia/macrophages suggests that inhibitors of CD40-TRAF2 signaling may effectively inhibit inflammation at the level of both non-hematopoietic and myeloid cells in DR.
